# SP8 Transcriptional Regulation of *Cyclin D1* During Mouse Early Corticogenesis

**DOI:** 10.3389/fnins.2018.00119

**Published:** 2018-03-02

**Authors:** Ugo Borello, Barbara Berarducci, Edwige Delahaye, David J. Price, Colette Dehay

**Affiliations:** ^1^Université de Lyon, Université Claude Bernard Lyon 1, Inserm, Stem Cell and Brain Research Institute U1208, Bron, France; ^2^Inovarion, Paris, France; ^3^Centre for Integrative Physiology, University of Edinburgh, Edinburgh, United Kingdom

**Keywords:** corticogenesis, gene expression regulation, *Cyclin D1*, transcription factors, SP8, PAX6

## Abstract

Multiple signals control the balance between proliferation and differentiation of neural progenitor cells during corticogenesis. A key point of this regulation is the control of G1 phase length, which is regulated by the Cyclin/Cdks complexes. Using genome-wide chromatin immunoprecipitation assay and mouse genetics, we have explored the transcriptional regulation of *Cyclin D1* (*Ccnd1*) during the early developmental stages of the mouse cerebral cortex. We found evidence that SP8 binds to the *Ccnd1* locus on exon regions. *In vitro* experiments show SP8 binding activity on *Ccnd1* gene 3′-end, and point to a putative role for SP8 in modulating PAX6-mediated repression of *Ccnd1* along the dorso-ventral axis of the developing pallium, creating a medial^Low^-lateral^High^ gradient of neuronal differentiation. Activation of *Ccnd1* through the promoter/5′-end of the gene does not depend on SP8, but on βcatenin (CTNNB1). Importantly, alteration of the *Sp8* level of expression *in vivo* affects *Ccnd1* expression during early corticogenesis. Our results indicate that *Ccnd1* regulation is the result of multiple signals and that SP8 is a player in this regulation, revealing an unexpected and potentially novel mechanism of transcriptional activation.

## Introduction

The cerebral cortex is the most complex structure of the mammalian brain. It is the site of numerous higher-order sensory, motor, and cognitive functions. Cortical function relies on the proper formation of specialized cortical areas as well as on their sophisticated interconnections (Glasser et al., 2016).

During development, regionalization of the embryonic brain is achieved through multi-step processes. Sources of diffusible signaling molecules act as organizing centers and pattern neighboring domains through regulation of specific transcription factors expression, thereby creating molecular compartments that lead to the generation of distinct cortical fields (Rubenstein et al., 1998; Sur and Rubenstein, 2005; O'Leary et al., 2007).

Cortical projection neurons are generated in the germinal zones (GZ) of the dorsal telencephalon and, following cell-cycle exit, migrate radially to the cortical plate. Previous work has shown that regional differences in the proliferative programs in the GZ have far reaching consequences for histogenesis of cortical areas (Dehay et al., 1993; Polleux et al., 1997; Lukaszewicz et al., 2005).

Neuron number and types specific of each cortical layer and area are defined by the fine-tuned balance between proliferation and differentiation of cortical progenitor cells. While cell biology mechanisms underlying the switch from proliferative to differentiative divisions have been identified (Fish et al., 2006; Delaunay et al., 2014, 2017; Mora-Bermudez et al., 2014; Paridaen and Huttner, 2014; Matsuzaki and Shitamukai, 2015), it has been shown that the increasing fraction of progenitor cells that quit the cell cycle to embark on neuronal differentiation correlate with a lengthening of the G1 phase of the cell cycle (Takahashi et al., 1995; Calegari et al., 2005; Salomoni and Calegari, 2010; Arai et al., 2011). G1 phase is considered as a time window of susceptibility to differentiation signals (Mummery et al., 1987) and G1 phase lengthening increases the competence of a proliferating cell to withdraw from the cell cycle and to differentiate (Zetterberg et al., 1995).

During corticogenesis, proliferative and differentiative divisions are characterized by short and long G1 phases respectively (Dehay et al., 2001; Lukaszewicz et al., 2002, 2005; Calegari and Huttner, 2003; Dehay and Kennedy, 2007; Pilaz et al., 2009). Progression through G1 phase is regulated mainly by the kinase activity of Cyclin D/CDK4 and Cyclin E/CDK2 (Sherr and Roberts, 2004), both of which have been shown to play a key role in determining neuron number during mouse mid-corticogenesis (Lange et al., 2009; Pilaz et al., 2009). In particular, *Cyclin D1* (*Ccnd1*) dynamic expression levels have been shown to be at the heart of a regulatory network that control the balance between cortical progenitor proliferation and differentiation (Ghosh et al., 2014).

Here we have explored the transcriptional regulation of *Ccnd1* expression during early corticogenesis. Numerous transcriptional factors binding to the *Ccnd1* promoter have been identified (Klein and Assoian, 2008). It is targeted by TCF/βcatenin complex (Shtutman et al., 1999; Tetsu and McCormick, 1999), effector of the Wnt pathway, which plays a key role in regulating cortical expansion (Chenn and Walsh, 2003). More recently, it has been reported that the transcription factor PAX6, known to regulate proliferation and differentiation of cortical progenitors (Warren et al., 1999; Estivill-Torrus et al., 2002; Quinn et al., 2007; Sansom et al., 2009; Mi et al., 2013) binds to the *Ccnd1* locus (Sun et al., 2015).

PAX6 plays a key role in forebrain patterning and cortex arealization (Stoykova et al., 1997; Bishop et al., 2000, 2002; Muzio et al., 2002; Englund et al., 2005). Interestingly, *Pax6* shows a complementary expression pattern to the transcription factor *Sp8* in the developing pallium with a rostro-ventral^High^ gradient (Sahara et al., 2007; Borello et al., 2014). SP8 is a zinc finger transcription factor belonging to the Sp-family of transcription factors (Zhao and Meng, 2005). SP8 acts downstream of FGF8 signaling (Storm et al., 2006), regulates forebrain patterning and cortical arealization (Sahara et al., 2007; Zembrzycki et al., 2007; Borello et al., 2014), and regulates cortical progenitor cell differentiation (Borello et al., 2014). Interestingly, SP5/SP8 have been shown to act as co-activators of the Wnt pathway in mouse embryos and differentiating embryonic stem (ES) cells (Kennedy et al., 2016).

We have therefore sought to analyze the putative role of SP8, together with PAX6 and βcatenin, on the transcriptional control of *Ccnd1*. Our ChipSeq and mouse genetics analysis reveal that *Ccnd1* is a target gene of SP8. We show that SP8 is a critical player in the regulation of *Ccnd1* expression during *in vivo* mouse corticogenesis. SP8 is able to modulate the moderate repressive transcriptional activity exerted by PAX6 on the *Ccnd1* exon 1 region *in vitro*. By contrast, we did not observe cooperation between SP8 and βcatenin on *Ccnd1* activation from the promoter/5′end of the gene. Finally, we demonstrate that SP8 is able to specifically activate gene expression from the *Ccnd1* exon 5 fragment, containing part of the 3′UTR, suggesting that the 3′-end of the *Ccnd*1 gene may be target of gene regulation at multiple levels, including transcription.

## Materials and methods

### Animals

*Foxg1*^*tTA*+/−^ and *tetO-Sp8-IE* mice (Waclaw et al., 2009), *Foxg1*^*cre*^ (Hebert and McConnell, 2000) and *Sp8*^*fl*/*fl*^ (Waclaw et al., 2006) mice were maintained and genotyped as already described (Waclaw et al., 2006, 2009, 2010). Mouse colonies were maintained at the SBRI/INSERM U1208, in accordance with the European requirement for animal experimentation 2010/63/UE. The protocol APAFIS #4748 has been approved by the Animal Care and Use Committee CELYNE (C2EA #42).

### Histology and *in situ* RNA hybridization (ISH)

Embryos were collected considering noon on the day of the vaginal plug as E0.5. The embryos were dissected and fixed overnight by immersion in 4% paraformaldehyde (PFA) in phosphate buffered saline (PBS) at 4°C. The tissue was cryoprotected by immersion in 30% sucrose/PBS, embedded in OCT (Tissue-Tek), and cryostat sectioned at 20 μm.

*In situ* RNA hybridization on cryostat sections was performed as previously described (Borello et al., 2008). cRNA probes used were: *Sp8* (K. Campbell, Cincinnati Children's Hospital, OH, USA), *Ccnd1* (A. Mallamaci, SISSA, Trieste, IT), *Axin2* (B. Cheyette, UCSF, USA), and *Pax6* (D. Price, University of Edinburgh).

Gene expression patterns were compared between brains of different genotypes by matching the plane of section according to multiple anatomical features. Whenever possible, this was performed for multiple planes of sections for each gene, and from at least three brains for each genotype.

*Foxg1*^*tTA*/+^ and the *tetO-Sp8-IE* mice were used as control; differences in phenotype were not observed between these two lines or between *Foxg1*^*tTA*/+^ and the *tetO-Sp8-IE* mice and the wild type embryos.

### ChipSeq

Dorsal telenchephalon (pallium) was dissected from E12.5 CD1 mouse embryos. The cells were crosslinked with 1% formaldehyde for 10 min. The formaldehyde reaction was quenched by adding glycine to a final concentration of 0.125 M for 10 min. Cells were then pelleted, rinsed once in cold phosphate-buffered saline (PBS) with 1 mM PMSF and once in cold lysis buffer (10 mM Tris pH 7.5, 10 mM NaCl, 3 mM MgCl2, 0.5% NP-40, and Roche Complete Protease Inhibitor Cocktail) to obtain nuclear pellets. Nuclei were sonicated in RIPA buffer (1X PBS, 1% NP-40 Substitute, 0.5% Sodium Deoxycholate, 0.1% SDS, and Roche Complete Protease Inhibitor Cocktail) at a concentration of 5 × 10^7^ nuclei/mL using a diagenode sonicator (Bioruptor Plus). The DNA fragments bound by SP8 were isolated using a goat polyclonal anti-SP8 antibody (C-18, Santa Cruz Biotechnology), a rabbit polyclonal anti-SP8 antibody (ab739494, abcam), or rabbit polyclonal H3K27ac (ab4729, abcam) coupled to magnetic beads (Dynabeads, ThermoFisher). The beads were washed 5 times with LiCl Wash Buffer (100 mM Tris pH 7.5, 500 mM LiCl, 1% NP-40, 1% sodium deoxycholate) and finally with TE buffer (10 mM Tris-HCl pH 7.5, 0.1 mM Na_2_EDTA).

The DNA was incubated o/n at 65°C in elution buffer (1% SDS, 0.1 M NaHCO_3_) to reverse the formaldehyde crosslink and was purified using a QIAquick PCR Purification Kit (Qiagen), following the manufacturer protocol. To check for fragment size distribution after sonication, a small fraction of the sample was reverse cross-linked for 2 h at 65°C, purified using DNA purification columns from Qiagen, then loaded onto a 2% agarose gel.

Sequence base calls were made using standard Illumina methods. Resulting 1 × 50 bp sequences were filtered to remove sequencing artifacts and adaptors and then mapped to the mouse genome (mm9) using the BWA algorithm (Li and Durbin, 2009). The resulting uniquely mapped reads were used for peak calling with MACS1.4 for SP8 and MACS2.1 for H3K27ac (Zhang et al., 2008; Feng et al., 2011), using recommended settings for transcriptional factor analysis and histone marks respectively. Called peaks were filtered to remove regions where a significant number of artifacts could originate (Consortium, 2012) (https://sites.google.com/site/anshulkundaje/projects/blacklists). Pearson's correlation on the two replicates calculated with a call to wigCorrelate (http://hgdownload.soe.ucsc.edu/admin/exe/macOSX.x86_64/) or Wiggletools (Zerbino et al., 2014) gave a value of 0.9. Peaks were annotated based on nearest transcription start site (TSS) using the Bioconductor package ChiPpeakAnno (Zhu et al., 2010) and ChiPseeker (Yu et al., 2015) and visualized using the Gviz package (Hahne and Ivanek, 2016).

The SP8 ChipSeq data presented in the “Results” section were obtained using the goat polyclonal anti-Sp8 antibody. These results were confirmed with a SP8 ChipSeq performed on two other independent biological replicates with the rabbit polyclonal anti-Sp8 antibody (Table S1 and data not shown).

### Cell transfection and luciferase assay

P19 cells (ATCC number: CRL-1825) were maintained in growth medium: Alpha Minimum Essential Medium with ribonucleosides and deoxyribonucleosides (ThermoFisher) completed with 7.5% bovine calf serum and 2.5% fetal calf serum (ThermoFisher) (McBurney and Rogers, 1982; McBurney et al., 1982). The cells were transfected with the expression vector for the full-length cDNA of human βcatenin (gifts of Dr Grosschedl, Max Planck Institute of Immunology, Germany), or *Pax6* (D. Price, University of Edinburgh, UK), or *Sp8* (gift of K. Campbell, Cincinnati Children's Hospital, OH, USA), along with the different *Ccnd1* fragments identified by ChipSeq (Table S1), cloned in the pGL4.10[Luc2] vector (Promega) containing the human β*globin* minimal promoter upstream of the luciferase gene (*Luc2, Photinus pyralis*). The fragment named *Ccnd1* exon 2.3 contains *Ccnd1* exons 2 and 3. The vector pG4.74[hRLuc/TK] (Promega), containing the Renilla luciferase gene, was co-transfected for normalization. The TK promoter of the pG4.74[hRLuc/TK] vector was substituted with the human β*globin* minimal promoter (from vector BGZ40) (Yee and Rigby, 1993).

The cells were transfected with Lipofectamine 2000 (ThermoFisher) in OPTIMEM medium (ThermoFisher) following the manufacturer's instructions, and cultured after 6 h in growth medium. Twenty-four hours after the transfection the cells were harvested in lysis buffer (Promega), and the luciferase and renilla luciferase activities were measured using the Dual Luciferase Assay protocol (Promega). Each transfection experiment was performed in triplicate and repeated at least two times. Reporter gene activities shown in **Figures 3–5** represent the average of the three replicates obtained in one representative transfection experiment. Statistical analysis was performed using the statistical package R and ANOVA analysis was performed using the “aov” R function and Tukey multiple comparison test. *p* < 0.05 were considered statistical significant.

## Results

### *Cyclin D1* is expressed in the developing forebrain at E12.5 with a rostro-ventral^High^ gradient

**Figure 1:**
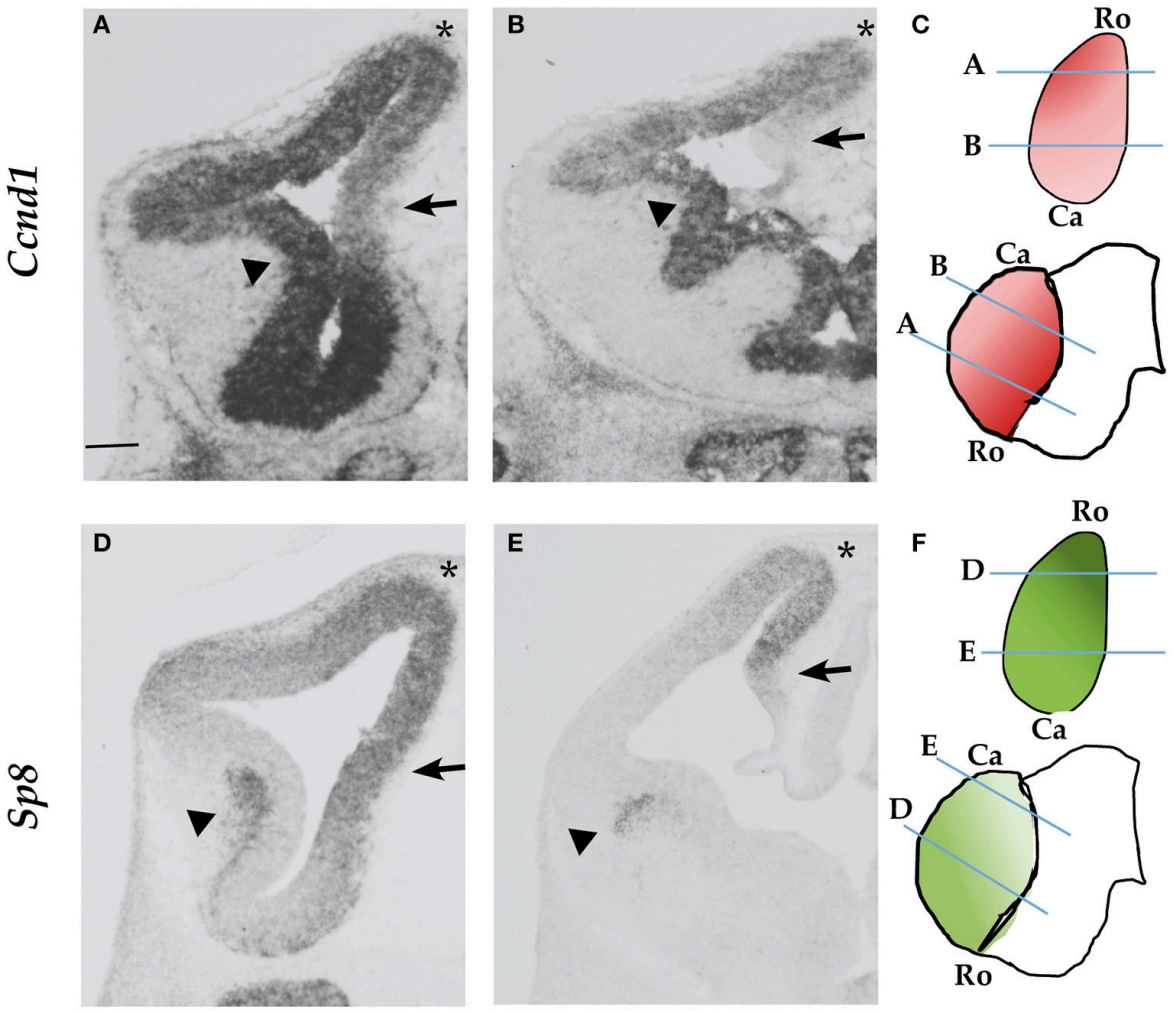
*Ccnd1* and *Sp8* expression patterns at E12.5. ISH performed on E12.5 mouse forebrain coronal sections. Panels **(A,B)** show *Ccnd1* mRNA expression; Panels **(D,E)** indicate *Sp8* mRNA expression. Schematic of *Ccnd1* **(C)** and *Sp8* **(F)** gradients of expression are indicated along with the positions of sections shown in **(A–D)**. Panels **(A,D)** represent sections at the rostral level, while Panels **(B,E)** represent sections at the caudal level. Arrows point to the medial pallium; arrowheads indicate the VZ **(A,B)** or the SVZ of the LGE **(D,E)**. The asterisk indicates the dorsal pallium. Bar in panel **(A)** is 200 μm. Ro, rostral; Ca, Caudal.

*Cyclin D1* is a key regulator of G1 phase progression in neural progenitor cells. We analyzed the mRNA expression of *Cyclin D1* in the embryonic forebrain at E12.5. We found that while *Ccnd1* is strongly expressed in the ventricular zone (VZ) of the basal ganglia (Figures 1A,B, arrowheads), its expression in the pallial VZ follows a rostro-lateral^High^ gradient (Figures 1A–C). In particular, *Ccnd1* expression is low in the medial pallium compared to dorsal and lateral regions (Figure 1A, arrow), while it is not expressed caudally in the hem (Figure 1B, arrow). This shows that *Ccnd1* is not expressed in all pallial progenitor cells at the same level, suggesting that the complex *Ccnd1* expression pattern is regulated by different factors.

*Sp8* is expressed in the pallium with a rostro-medial^High^ gradient (Figures 1D–F). *Sp8* is expressed in the pallial VZ, as *Ccnd1*. In the subpallium *Sp8* is expressed in the subventricular zone (SVZ) of the lateral ganglionic eminence (LGE) (Figures 1D,E, arrowheads), while *Ccnd1* is expressed in the subpallial VZ (Figures 1A,B, arrowheads).

*Ccnd1* appears to be highly expressed in the dorsal pallium were *Sp8* expression is high (Figures 1A,B,D,E, asterisks); interestingly *Ccnd1* expression is lower in the medial pallium, a region of strong *Sp8* expression (Figures 1A,B,D,E, arrows).

In conclusion, the expression pattern of *Sp8* is compatible with the possibility that it contributes to the transcriptional regulation of *Ccnd1* in the dorso-medial pallium.

### The zinc finger transcriptional factor SP8 binds to the *Ccnd1* locus in cortical progenitor cells

**Figure 2:**
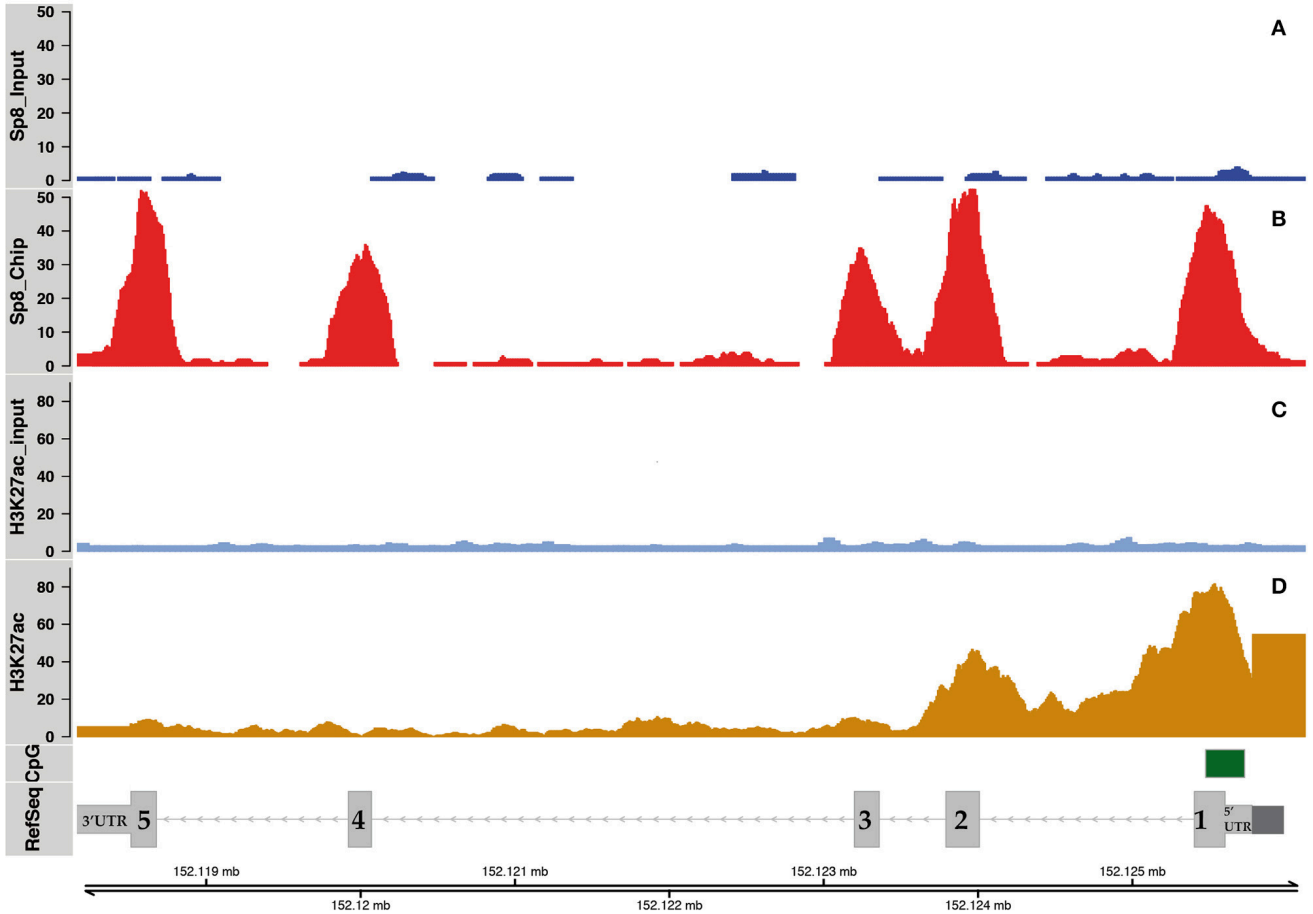
SP8 binding regions on the *Ccnd1* locus. Panels **(A,B)** show SP8 ChipSeq peaks in input and SP8 antibody (Ab) treated samples respectively. Panel **(C)** shows normalized H3K27ac ChipSeq peaks. Panel **(D)** shows *Ccnd1* RefSeq gene model in light gray, the promoter (Eto, 2000) in dark gray, and the CpG islands in green. *Sp8* exons are numbered and the UTRs are indicated.

To test the hypothesis that SP8 regulates *Ccnd1* at the transcriptional level, we performed SP8 ChipSeq experiments using E12.5 mouse embryos pallial cells (manuscript in preparation). We found that SP8 binds the *Ccnd1* locus *in vivo* decorating *Ccnd1* exons (Figures 2A,B), with higher values for exon 1 containing the 5′UTR, exon 2, and exon 5 containing part of the 3′UTR.

The presence of acetylated histone H3 lysine 27 (H3K27ac) on exons 1 and 2 indicated that these regions correspond to active chromatin domains (Figures 2C,D). The *Ccnd1* exon 5 and exon 3 co-localize with the H3K27ac signal, even though it is of smaller intensity than in the exon 1 (Figures 2C,D). H3K27ac signals were obtained from ChipSeq experiments using, as for SP8, E12.5 mouse pallial cells (data not shown, manuscript in preparation).

The fact that *Ccnd1* 5′UTR showed H3K27ac signal and it contains a CpG island suggests a role for this region in the transcriptional regulation of *Ccnd1* in E12.5 cortical progenitor cells (Figures 2C,D). Moreover, *Ccnd1* promoter and the 3′UTR represent important regulative regions for the transcriptional regulation of this gene (Klein and Assoian, 2008; Deshpande et al., 2009; Guo et al., 2011). Together these data indicate that SP8 binds transcriptionally active regions in the *Ccnd1* locus *in vivo* in pallial progenitor cells.

### SP8 regulates gene expression through *Ccnd1* exon 5 fragment *in vitro*

The observation that SP8 binds mainly on *Ccnd1* exons is intriguing. It is generally assumed that the coding genome is physically distinct from the regulatory genome. Consequently, the binding of transcription factors to gene exons is considered generally non-functional (Li et al., 2008). Therefore, we evaluated the relevance of SP8 binding on *Ccnd1* exons observed in our ChipSeq experiments.

To test the transcriptional activity of SP8 on the different *Ccnd1* exons we performed a luciferase assay *in vitro*. We focused on the *Ccnd1* exons showing both SP8 ChipSeq peaks and active chromatin signature (i.e., H3K27ac signal) (Figure 2). The exon 1 (Ex1) fragment contained the last 293 bp of the *Ccnd1* promoter (Eto, 2000), the entire exon 1 (containing the 5′UTR) and the first 526 bp of intron 1 (Figure 2 and Figure S1). The exon 5 (Ex5) fragment spanned from intron 4 (last 425 bp) to the coding region up to the first 299 bp of the 3′UTR (Figure 2 and Figure S2).

**Figure 3:**
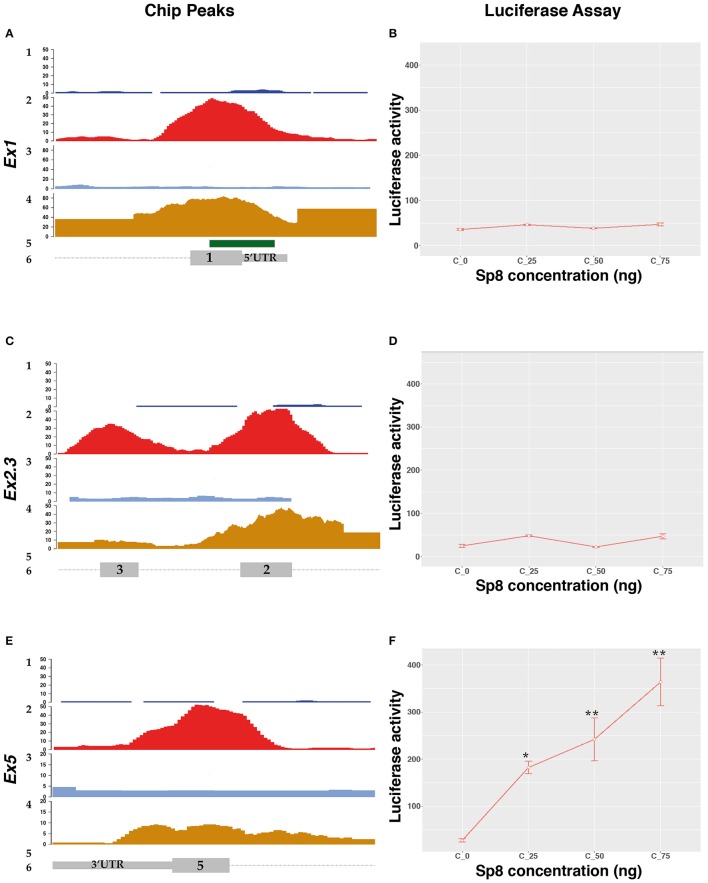
SP8 transcriptional regulation of *Ccnd1* genomic regions bound by Sp8. Panels **(A,C,E)** show details of the *Ccnd1* locus where the SP8 ChipSeq peak have been detected: **(A)** SP8 peak at the *Ccnd1* Ex1 fragment; **(C)** SP8 peak at the *Ccnd1* Ex5 fragment; **(E)** Sp8 peak at the *Ccnd1* Ex2.3 fragment. 1: SP8 Chip input peaks, 2: SP8 Chip peaks, 3: H3K27ac Chip Input peaks, 4: H3K27ac Chip peaks, 5: CpG island, 6: *Ccnd1* RefSeq gene model. For details refer to the legend of Figure 2. Panels **(B,D,F)** show the results of the luciferase assays performed with *Ccnd1* Ex1 **(B)**, *Ccnd1* Ex2.3 **(F)**, and *Ccnd1* Ex5 **(D)** fragments. Statistical significance, ANOVA: ^*^p < 0.05, ^**^p < 0.01.

The DNA region corresponding to the SP8 ChipSeq peaks were cloned upstream of the luciferase gene and tested in P19 cells in the presence of increasing levels of SP8. Surprisingly, we found that SP8 had no activity on the *Ccnd1* Ex1 fragment (Figures 3A,B), nor on exons 2 and 3 (Ex2.3) fragment (Figures 3C,D). However, increasing amounts of SP8 activated the luciferase gene through the *Ccnd1* Ex5 fragment (Figures 3E,F).

Bioinformatic analysis using the Jaspar software, indicated 7 putative SP8 binding sites located in the Ex5 fragment (Table S2); specifically, a cluster of 6 sites is located in the exon 5 ORF (Figure S2). This region contains the SP8 peak summit identified in our ChipSeq results (Figure 5 and Figure S2). This unexpected result indicates that SP8 binds to the exonic region 5 of *Ccnd1*, thereby modulating its transcription.

### βcatenin and PAX6 regulate *Ccnd1* exon1 fragment activity *in vitro*

The Wnt/βcatenin pathway was demonstrated to be a major regulator of *Ccnd1* gene expression (Shtutman et al., 1999; Tetsu and McCormick, 1999). The Wnt pathway regulates gene expression by binding of the cofactor βcatenin to genomic regulative regions specifically recognized by TCF/LEF, the effectors of the Wnt pathway (Clevers, 2006; van Amerongen and Nusse, 2009).

**Figure 4:**
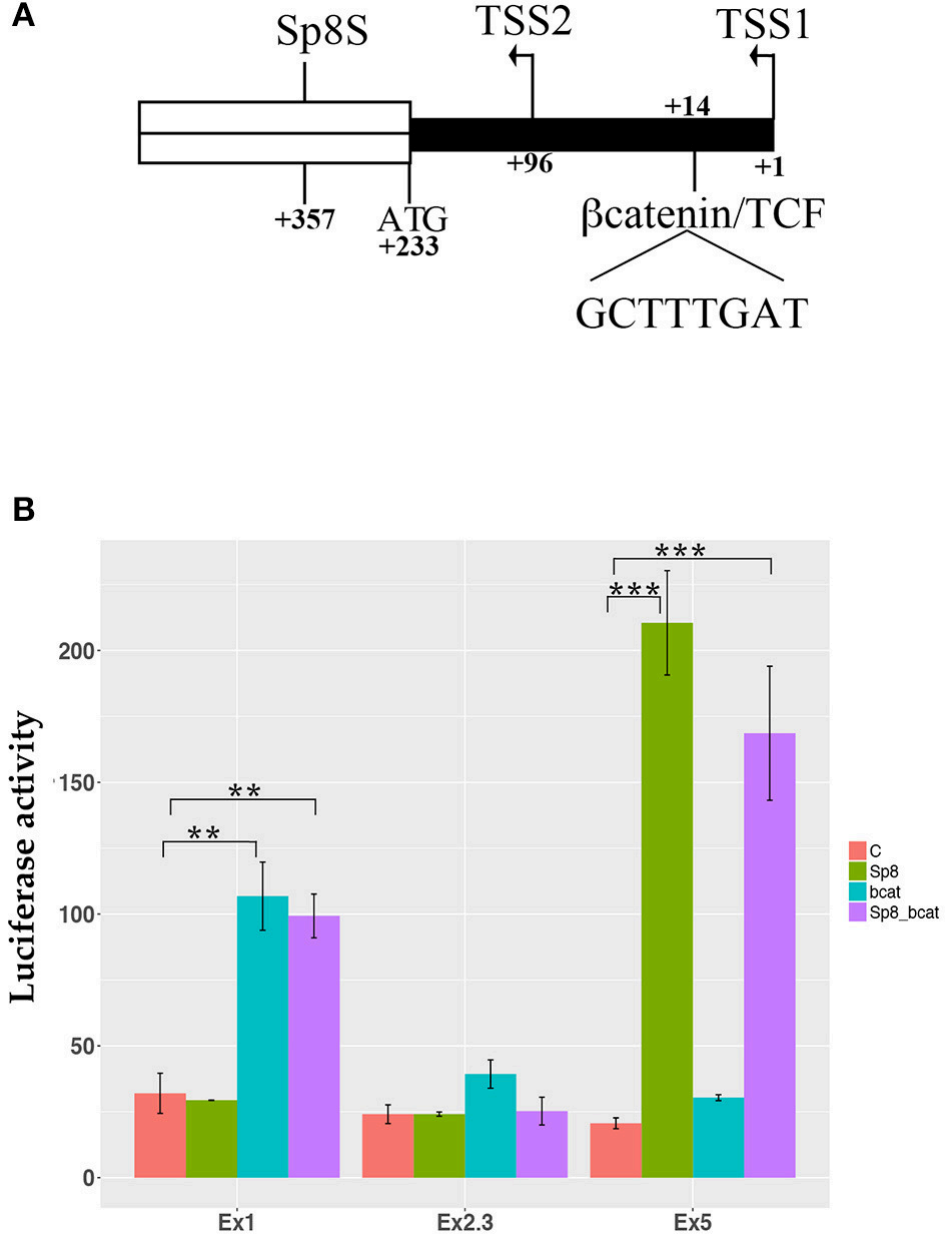
βcatenin and PAX6 transcriptional regulation of *Ccnd1* regulative regions identified by SP8 ChipSeq. **(A)** represents the schema of the 5′ portion of the exon 1 fragment. The transcriptional start sites, TSS1 and TSS2, the first codon ATG, and the conserved βcatenin binding site (Klein and Assoian, 2008), and the SP8 summit (SP8S) positions are indicated. The 5′UTR region is depicted in black; the ORF of exon 1 is indicated in white. Panel **(B)** shows the results of the luciferase assay obtained from one representative experiment. P19 cells were transfected with the *Ccnd1* exon fragments identified by SP8 ChipSeq alone or in combination with βcatenin, SP8, or βcatenin together with SP8. Statistical significance, ANOVA: ^**^p < 0.01, ^***^p < 0.001.

Interestingly, the SP8 ChipSeq peak corresponding to exon 1 and containing the last 293 nucleotides of the mouse *Ccnd1* promoter (Eto, 2000), contains a highly conserved consensus for TCF/LEF transcriptional factors (Klein and Assoian, 2008) (Figure 4A). As in human, mouse *Ccnd1* promoter has no TATA or TATA-like sequence, and the TSS is determined by the Initiator sequence (Inr) (Eto, 2000). However, two possible Inr sequences are present in the mouse *Ccnd1* promoter, the second one located at nt +90 from the first Inr sequence, determining a TSS at nt +96 (Eto, 2000) (Figure 4A). According to the Inr site described by Eto (2000), the conserved TCF/LEF site is located downstream to the first TSS, starting at nt + 14, in the *Ccnd1* 5′UTR (Figure 4A).

We tested βcatenin transcriptional activity in combination with SP8 on the above described *Ccnd1* fragments. We used a constitutively active form of βcatenin that is not degraded by the proteasome and accumulates into the nucleus (Hsu et al., 1998) in a luciferase assay *in vitro*. Our results showed that βcatenin activates luciferase transcription specifically through the *Ccnd1* Ex1 fragment, and that SP8 does not modulate this effect (Figure 4B). No effect was observed on *Ccnd1* Ex2.3 or Ex5 fragments (Figure 4B).

**Figure 5:**
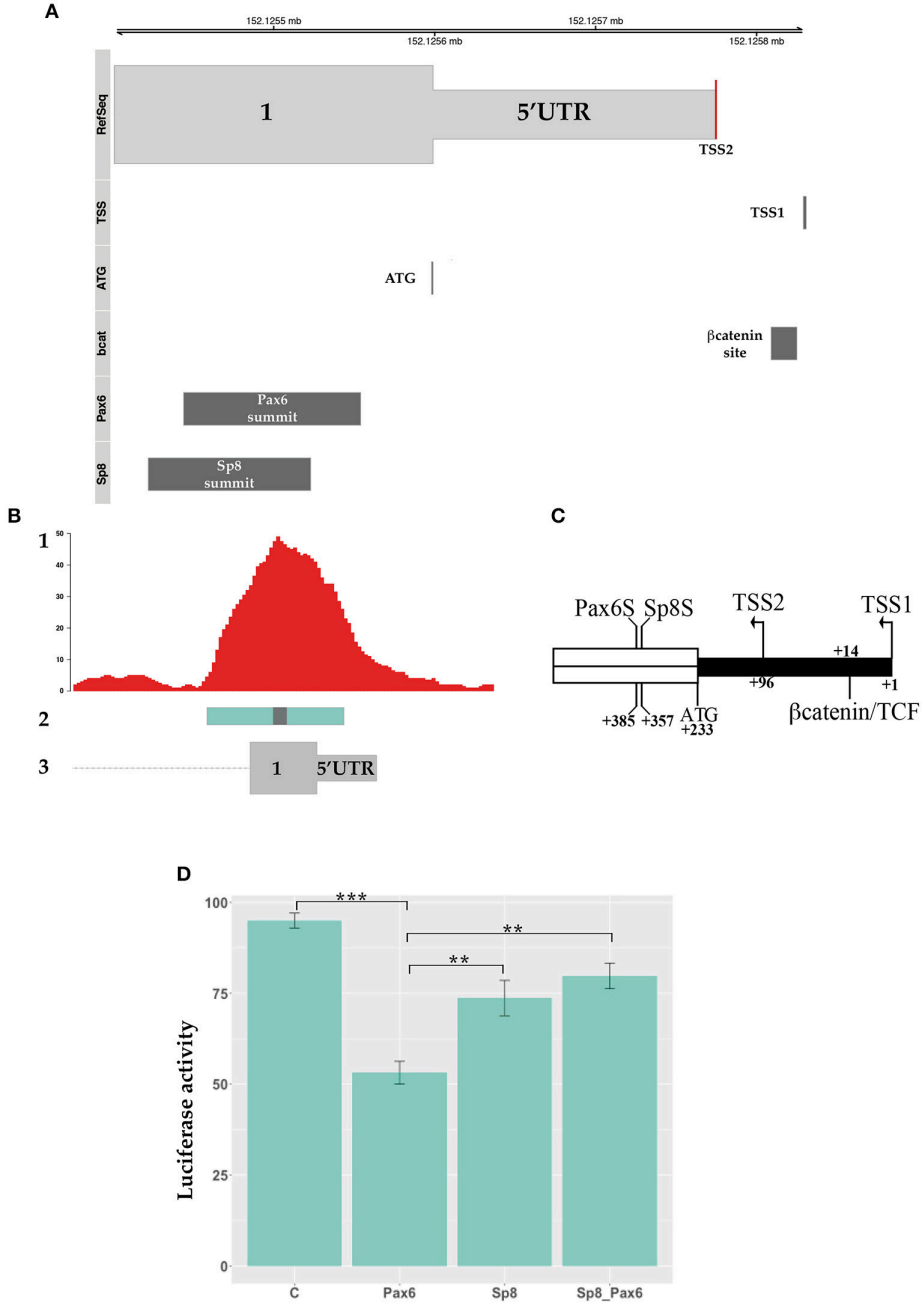
PAX6 transcriptional regulation of *Ccnd1* regulative regions. Panel **(A)** shows the schema of *Ccnd1* locus. PAX6 [Chip peak fragment indicated in Sun et al. (2015)], SP8 (Chip peak summit ±50 bp), βcatenin binding sites, and the TSS (named TSS1) described in Eto (2000) are depicted together with *Ccnd1* exon 1 first codon (ATG) and the TSS reported by the RefSeq gene model (TSS2, red line on the RefSeq track). Panel **(B)** represents the schema of the SP8 and PAX6 peaks positions on the *Ccnd1* locus, showing the overlap between the SP8 (this work) and PAX6 ChipSeq peaks (Sun et al., 2015) on *Ccnd1* Ex1 fragment. 1: SP8 Chip peaks, 2: PAX6 chipped fragment, 3: *Ccnd1* RefSeq gene model. The gray box on the *Pax6* fragment is the PAX6 binding summit shown in **(A)**. Panel **(C)** shows a schematic of βcatenin, SP8 and PAX6 sites position on the 5′ portion of the exon 1 fragment. The PAX6 (PAX6S) and SP8 (SP8S) summit positions [indicated as the central nt of the Chip-qPCR fragment indicated in Sun et al. (2015) and the calculated ChipSeq summit respectively] are indicated. The TSS described in Eto (2000) (TSS1), the *Ccnd1* first codon (ATG), and the TSS reported by the RefSeq gene model (TSS2) are also shown. The 5′UTR is depicted in black. Panel **(D)** shows the luciferase assay results obtained from one representative experiment. *Ccnd1* exon 1 was transfected in P19 cells alone or in combination with PAX6, SP8, and PAX6 together with SP8 (SP8_PAX6). Statistical significance, ANOVA: ^***^p < 0.001, ^**^p < 0.01.

PAX6 is a transcription factor which regulates forebrain patterning and growth. It is expressed with a complementary gradient to that of *Sp8* (Figures S3A,B). PAX6 binds to the *Ccnd1* locus (Sun et al., 2015). When we compared the position of the PAX6 ChipSeq peak with that of SP8, we found that the two transcription factors bind to an overlapping region in the Ex1 fragment and that the SP8 peak summit was located near the PAX6 binding region (Figures 5A,B and Figure S1). Bioinformatics analysis using the Jaspar database showed a potential PAX6 binding site in the Ex1 fragment at position + 392 from the first TSS (Figure 5C and Figure S1); this predicted consensus sequence is near the summit of the PAX6 ChipSeq peak (Figure S1) (Sun et al., 2015).

We tested the effect of PAX6 on *Ccnd1* Ex1 fragment transcriptional activity and the effect of SP8 upon co-expression. Our results showed that PAX6 exerts a moderate repressive transcriptional activity on the *Ccnd1* exon 1 region, and that SP8 counteracts this repression when co-transfected with PAX6 (Figure 5D).

### SP8 regulates *Ccnd1* during *in vivo* corticogenesis

To further test the role of SP8 on *Ccnd1* gene regulation we analyzed the relevance of our *in vitro* results by altering the level of *Sp8* expression *in vivo* during corticogenesis. For this purpose, we took advantage of genetic systems in which *Sp8* was either overexpressed or absent. In the *Sp8* gain-of-function (GOF) transgenic mouse system, *Sp8* is over-expressed during forebrain development (Waclaw et al., 2009; Borello et al., 2014), while in the loss-of-function (LOF) transgenic mouse system (Waclaw et al., 2006; Borello et al., 2014), *Sp8* expression is eliminated (Figure S4).

**Figure 6:**
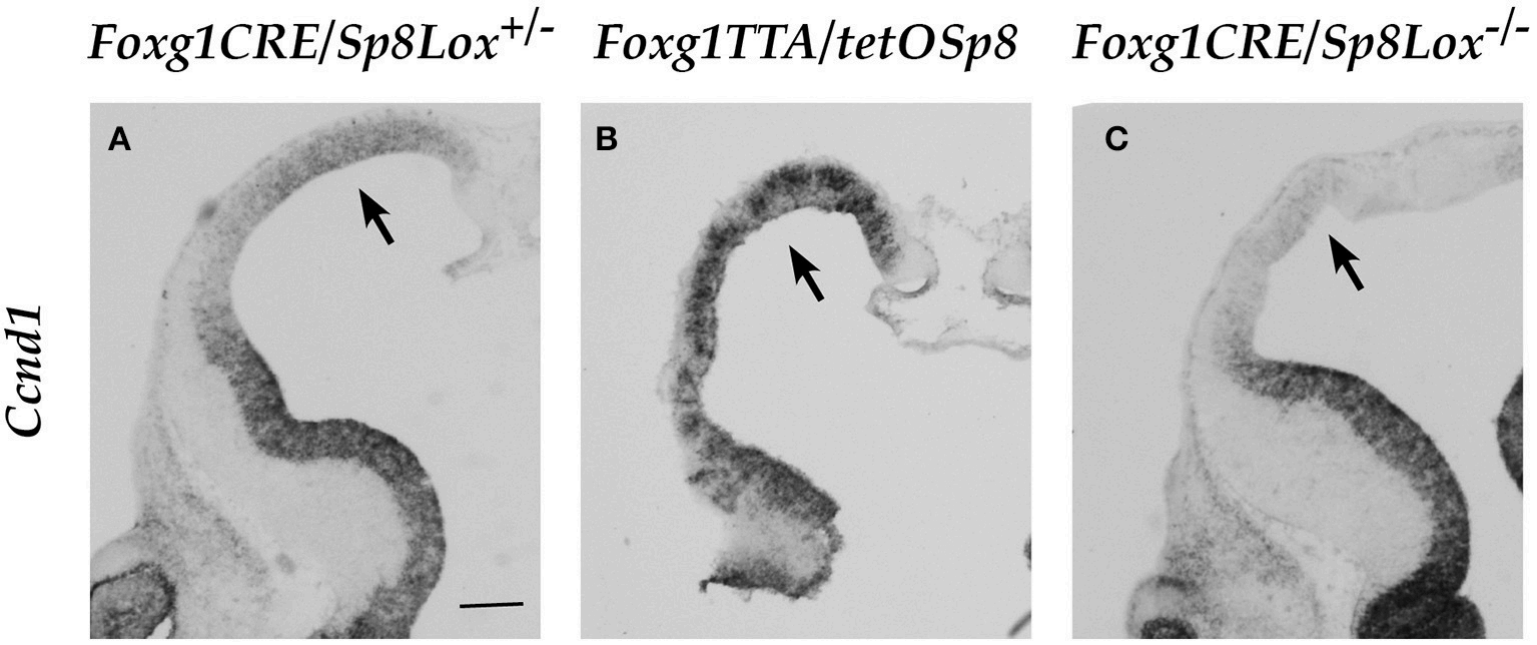
Expression of *Ccnd1* in embryonic forebrain after *Sp8* misexpression *in vivo*. ISH on E12.5 mouse forebrain coronal sections. Arrow in Panel **(A)** shows *Ccnd1* expression in the control pallial VZ. Arrows in Panels **(B,C)** show *Ccnd1* expression in the *Sp8* GOF and *Sp8* LOF pallial VZ respectively. Bar in **(A)**: 200 μm.

When we analyzed *Ccnd1* expression during early corticogenesis using these genetic tools we found that *Ccnd1* expression was strongly increased after *Sp8* over-expression in the GOF mutant mice (Figures 6A,B), while it was strongly reduced in the LOF mutant mice in regions corresponding to the higher *Sp8* expression domain, i.e. the rostral dorso-medial pallium (Figures 6A–C).

These data were further confirmed by RNASeq experiments (data not shown) performed on E12.5 mouse pallial cells showing an increase of *Ccnd1* expression in the *Sp8* GOF mutants of 3 folds (FDR adjusted *p* < 0.001) and a reduction of 0.8 folds in the *Sp8* LOF mutants (FDR adjusted *p*-value 0.09) (Table S3).

These findings indicate that SP8 is a critical player in the regulation of *Ccnd1* expression during mouse corticogenesis *in vivo*.

## Discussion

*Cyclin D1* is a major cell-cycle regulator (Ekholm and Reed, 2000; Sherr and Roberts, 2004) and has been shown to be at the heart of a regulatory network controlling the balance between proliferation and differentiation in the cerebral cortex (Ghosh et al., 2014).

βcatenin is one of the main regulators of *Ccnd1* expression (Shtutman et al., 1999; Tetsu and McCormick, 1999; Klein and Assoian, 2008). Our observations that *Ccnd1* expression signal does not necessarily correlate to regions of high Wnt/βcatenin activity during early *in vivo* corticogenesis is consistent with the idea that activation of the *Ccnd1* gene might be modulated by cooperation with other transcription factors. Indeed, *Axin2*, a direct target and recognized proxy of the Wnt/βcatenin pathway activity (Yan et al., 2001; Jho et al., 2002; Lustig et al., 2002; Kim et al., 2007; Al Alam et al., 2011; van Amerongen et al., 2012; Bowman et al., 2013), is strongly localized in the medial pallium where *Ccnd1* expression is low or absent and weakly expressed in the dorsal pallium where *Ccnd1* is highly expressed (Figures 1A,B and Figures S3C,D). Altogether these data suggest that while βcatenin regulates *Ccnd1* expression during corticogenesis *in vivo*, other transcription factors are also at work to produce the observed *Ccnd1* expression pattern in the dorso-medial pallium.

*Sp8* and *Ccnd1* expression patterns in the early mouse corticogenesis *in vivo* are consistent with a potential role of SP8 on *Ccnd1* gene regulation. The present data confirm this hypothesis and show the identification of *Ccnd1* as the first SP8 target gene.

SP8 binds on the *Ccnd1* locus on regions of active chromatin, as indicated by the H3K27ac ChipSeq results. Interestingly, peaks with higher intensity were positioned at the promoter/exon1 region, and in exon 5, containing also the first 299 bp of the 3′UTR. Our findings were further confirmed by results from SP8 ChipSeq experiments using a second SP8 antibody (Table S1 and data not shown). When we tested the responsiveness of these regions to SP8 we found that SP8 was able to activate gene expression from the *Ccnd1* Ex5 but not from the Ex1 fragment, containing the last 293 nucleotides of the *Ccnd1* promoter.

These results are unexpected. The regulatory regions of the genome are generally considered to localize outside of the coding sequences to keep the regulatory and the coding codes separated. However, a theoretical study predicts that the human genome, compared to a synthetic string of DNA letters, could accommodate short functional regulatory motifs in the protein coding regions (Itzkovitz and Alon, 2007). In addition, different studies aimed at identifying regulatory regions in the genome found that a small percentage of these regulatory domains are located in the coding sequences (Cawley et al., 2004; Visel et al., 2009) and that they are functional (Ritter et al., 2012). Recently, a comprehensive study mapping transcription factor binding on human genome exons in many cells lines found that ~15% of human codons specify both amino acids and transcription factor binding sites (Stergachis et al., 2013). Stergachis and colleagues suggest the fascinating hypothesis that the transcription factors binding to conserved sequences inside a gene exons have a role in codon choice and protein evolution. Numerous studies report that intergenic regulative regions like enhancers are sites of active transcription (De Santa et al., 2010; Kim et al., 2010; Natoli and Andrau, 2012; Shlyueva et al., 2014; Kim and Shiekhattar, 2015; Li et al., 2016), blurring the distinction between transcribed gene regions and regulative domains.

The Sp-family transcription factors bind preferentially GC and/or GT-reach regions in TATA-containing and TATA-less promoters and stimulate transcription by associating with the basal transcription complex and other transcription factors (Lania et al., 1997; Philipsen and Suske, 1999; Zhao and Meng, 2005). Consistently, the SP8 ChipSeq experiments showed that 78% of SP8 peaks correspond to gene promoters while genome wide SP8 binds only ~2% of gene exons and UTRs (see Figure S5 for details on the genome-wide SP8 binding localization). Interestingly, while our bioinformatics analysis identified several SP8 binding sites in the *Ccnd1* promoter contained in the Ex1 fragment (Table S4 and Figure S1), the ChipSeq experiment indicates that the SP8 summit is located in the coding region. We hypothesize that SP8 binding on *Ccnd1* exons is related to the fine-tuned regulation of *Ccnd1* transcription, probably through a precise chromatin 3D structure, as well as to *Ccnd1* mRNA maturation. There is also the possibility that SP8 is part of an epigenetic complex regulation of *Ccnd1* locus replication and transcription. These questions will require further investigations.

Of interest, PAX6, which generally colocalizes with enhancers, binds *Ccnd1* exon 1 (Sun et al., 2015). The predicted PAX6 binding site starts at position + 392 from the TSS (166 nucleotides downstream to the ATG), (Figures 2, 5A–C and Figure S1). Interestingly, while SP8 failed to directly regulate *Ccnd1* Ex1 fragment expression, we show that SP8 was able to counteract the repressive activity exerted by PAX6 on the *Ccnd1* Ex1 fragment *in vitro*. Moreover, the repressive activity we observed with PAX6 is consistent with the moderate increase of *Ccnd1* mRNA observed in the *Pax6* LOF E12.5 mutant forebrain (Mi et al., 2013; Sun et al., 2015).

SP8 could, therefore, interfere with PAX6 effect on *Ccnd1* expression. It is possible that, due to the close proximity of the SP8 and PAX6 consensus, the two transcription factors compete for the binding on *Ccnd1* exon 1. As mentioned above, *Sp8* and *Pax6* show opposite gradient of expression during early corticogenesis. At mid-gestation when *Pax6* expression becomes homogeneous in the pallium, *Sp8* expression is expressed at low levels. These data suggest that *Ccnd1* is activated differentially by *Sp8* and *Pax6* in opposite domains of the pallium and is modulated by PAX6 and SP8 dosages along the neurogenic gradient.

**Figure 7:**
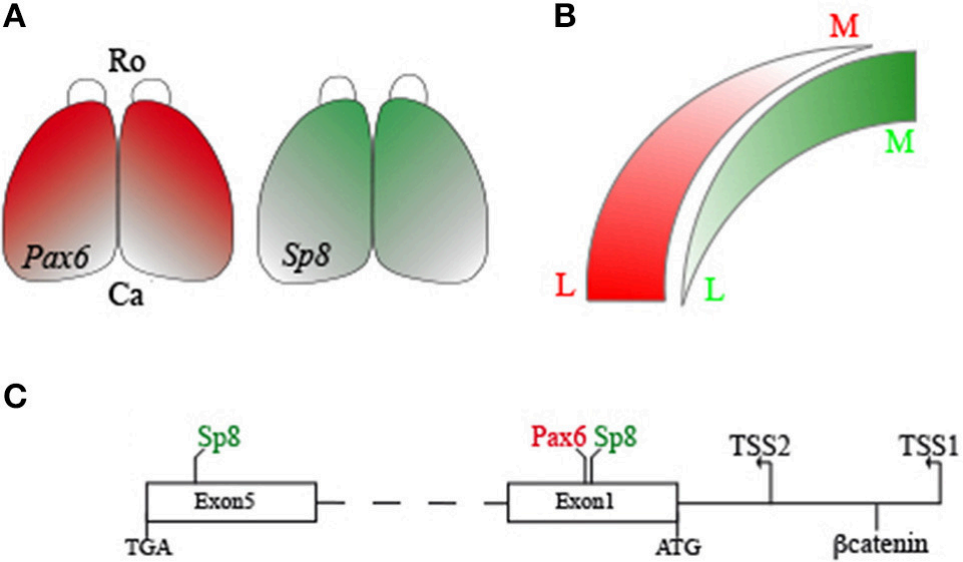
Schema of the SP8, PAX6, and βcatenin interactions on the mouse *Ccnd1* gene. Panel **(A)** shows *Sp8* and *Pax6* expression gradients in the developing mouse cortex. Panel **(B)** summarizes differences in *Sp8* and *Pax6* expression levels along the medio-lateral axis of the pallium. Panel **(C)** is a graphical representation of SP8, PAX6, and βcatenin binding positions on *Ccnd1* locus. R, Rostral; C, Caudal; M, Medial; L, Lateral.

SOX2 activates *Ccnd1* in a dose-dependent manner during corticogenesis (Hagey and Muhr, 2014). SOX2, binding on different sites on the *Ccnd1* locus and interacting with the TCF/βcatenin complex, regulates *Ccnd1* expression and cortical progenitor cell mode of division and rate of differentiation (Hagey and Muhr, 2014). Considering the *Sp8* graded expression in the pallial VZ and the strong *Sp8* expression in the subpallial SVZ (Waclaw et al., 2006; Borello et al., 2014), one can hypothesize that a dose-dependent differential transcriptional regulation is also operant for SP8 (Figure 7).

In contrast to PAX6, βcatenin was able to activate transcription from the *Ccnd1* 5′UTR, and this activation was not dependent or modulated by SP8. The mouse *Ccnd1* Ex1 fragment described here contains a TCF/LEF consensus that is conserved among different species, including human (Klein and Assoian, 2008), suggesting a critical and fundamental role for this site in *Ccnd1* regulation. In addition, Tetsu and colleagues showed that activation of the *CCND1* human minimal promoter,−962CD1 (Albanese et al., 1995), by βcatenin depends on the presence of TCF binding sites but not of other transcription factors (Tetsu and McCormick, 1999). These observations are in agreement with our results showing that the exon 1 fragment, containing only the last 293 nucleotides of the mouse *Ccnd1* promoter, was sufficient to support βcatenin activity. The fact that βcatenin activity was independent of SP8 indicates that these two transcription factors do not cooperate by binding the *Ccnd1* exon 1 region. However, a potential cooperation between βcatenin and SP8 binding to different *Ccnd1* exon fragments (i.e., exon 5) needs further investigation.

Our results show that SP8 is able to specifically activate gene expression from the *Ccnd1* Ex5 fragment. Consistently with our luciferase results, we found a cluster of putative SP8 binding sites at the end of the ORF in Ex5 fragment; this cluster overlapped with the SP8 summit identified in our ChipSeq experiments (Table S1 and Figure S2). These findings are very interesting as they rise the possibility that SP8 might control gene expression from binding to regions located at the 3′ end of the *Ccnd1* gene in addition to the classical enhancer/promoter regulative domains located upstream of the target genes.

Human *CCND1* 3′UTR region has been shown to act as a critical regulatory element. Different miRNAs are predicted to bind human and mouse *Ccnd1* 3′UTR and regulate the level of *Cyclin D1* expression (Deshpande et al., 2009; Ghosh et al., 2014); truncation or mutation of human *CCND1* 3′UTR alter the stability of the *CCND1* transcript activating its oncogenic potential (Lebwohl et al., 1994; Molenaar et al., 2003; Wiestner et al., 2007; Deshpande et al., 2009; Ghosh et al., 2014). In addition, different Snps are present in the 3′UTR of mouse and human *CCND1*: Snp rs7178, localized on *CCND1* 3′UTR, is involved in neuroblastoma (Wang et al., 2011), and Snp rs7177, localized on *CCND1* 3′UTR, is involved in cognitive behavior (Rietveld et al., 2013). Considering that there is a 78.1% identity between human and mouse *Ccnd1* 3′UTR (as revealed using the ECR Browser Ovcharenko et al., 2004), these observations suggest a similar role in gene regulation and neurogenesis for the mouse *Ccnd1* 3′UTR.

Our data, showing that SP8 binds and specifically regulates *Ccnd1* transcription from a region located at the end of the ORF in exon 5 and close to the 3′UTR, suggest that the 3′-end of the *Ccnd1* gene may be a target of gene regulation at multiple levels, including the transcriptional one. The *in vitro* validation of the activity of SP8, as well as the interaction with PAX6 and βcatenin on the *Ccnd1* locus, is based on an assay commonly used to screen the activity of genomic regulative regions. In addition, we provide further evidence based on manipulation of SP8 levels of expression *in vivo* in GOF and LOF transgenic mice as well as on RNAseq data that both clearly show a role for SP8 for *Ccnd1* expression regulation at early stages of pallium development.

In summary, multiple signals regulate *Ccnd1* transcription in mouse pallium during corticogenesis, resulting in a complex pattern of *Ccnd1* expression. SP8 appears as a major player in this regulation, uncovering a potential novel role of the Sp-transcription factor family in transcription regulation, which awaits further analysis.

## Author contributions

UB: Conceived the work; UB, BB, and ED: Collected and analyzed the data; UB, BB, DP, and CD: Wrote the paper.

### Conflict of interest statement

The authors declare that the research was conducted in the absence of any commercial or financial relationships that could be construed as a potential conflict of interest.

## Supplementary material

Figure S1 Predicted SP8 binding sites on *Ccnd1* Ex1 fragment. The predicted SP8 sites from Table S4 (A–G), the SP8 summit (SP8S), the PAX6 summit (PAX6S), the predicted PAX6 binding site, and the ATG are indicated. The nt positions refer to the *Ccnd1* TSS1; SP8 **(G)** site position is indicated as 164 nt downstream of the exon 1 ORF. The SP8 A-E sites are located in the promoter region; SP8 **(F,G)** sites are the closest to the SP8 summit. Positions of the SP8S and PAX6S are indicated in bold and underlined.

Figure S2 Predicted SP8 binding sites on *Ccnd1* Ex5 fragment. The 7 predicted SP8 sites from Table S2 (A–G), the SP8 summit (SP8S), and the stop codon (TGA) are indicated. The nt positions refer to the *Ccnd1* TSS1; SP8 **(A,B)** position is indicated as 125 nt upstream of the exon 5 ORF. Position of the SP8S is indicated in bold and underlined.

Figure S3 *Pax6* and *Axin2* expression at E12.5. ISH on E12.5 mouse forebrain coronal sections. Panel **(A)** shows *Pax6* expression in the rostral forebrain and panel **(B)** shows *Pax6* expression in a more caudal section, panel **(C)** shows *Axin2* expression, as proxy of the Wnt pathway activity, in the rostral forebrain and panel **(D)** shows *Axin2* expression in a more caudal section. Bar in **(A)**: 200 μm.

Figure S4 *Sp8* expression analysis in the *Sp8* LOF and GOF mutants at E12.5. E12.5 mouse forebrain coronal sections. *Sp8* mRNA expression levels are shown in the control **(A)** and *Sp8* LOF mutant **(C)**, immunofluorescence of EGFP **(B)** is shown as a proxy of *Sp8* overexpression in the GOF mutant (Borello et al., 2014). Bar in **(A)**: 200 μm.

Figure S5 Genome-wide distribution of the SP8 binding sites on gene features. Plot showing the percentage of the SP8 binding sites distributed genome-wide on gene features.

Table S1 SP8 and H3K27ac ChipSeq fragments identified on the *Ccnd1* locus. MACS results of the SP8 and H3K27ac ChipSeq peak calling. Position of the peaks summits is indicated. The column “name” indicates the genomic fragment names used in this study. The fragment named Ex1.2.3 in the H3K27ac ChipSeq dataset contains *Ccnd1* promoter, 5′UTR, and exons 1–3; fragment Ex5 contains *Ccnd1* exon 5 and 3′U TR.

Table S2 Bioinformatic analysis using the Jaspar software (Mathelier et al., 2016) of the *Ccnd1* Ex5 fragment. Position of the predicted SP8 sites refers to the Ex5 fragment full sequence, nt 1-889.

Table S3 *Ccnd1* expression levels in the *Sp8* GOF and LOF mutants. Results of RNASeq analysis on *Sp8* mutants obtained with DESeq2 (Love et al., 2014).

Table S4 Bioinformatic analysis of the *Ccnd1* Ex1 fragment using the Jaspar software (Mathelier et al., 2016). Position of the predicted SP8 sites refers to the Ex1 fragment full sequence, nt 1-1249. In bold are indicated the SP8 predicted sites in the *Ccnd1* promoter (SP8 **A–E)** showing the highest score values, and the two SP8 sites **(F,G)** close to the position of the SP8 summit. The position of the predicted PAX6 site is indicated.
