# Supplementary figures and images for: SP8 Transcriptional Regulation of Cyclin D1 During Mouse Early Corticogenesis
Source: Front Neurosci. 2018 Mar 2;12:119. doi: 10.3389/fnins.2018.00119 (PMC5863514; doi:10.3389/fnins.2018.00119)

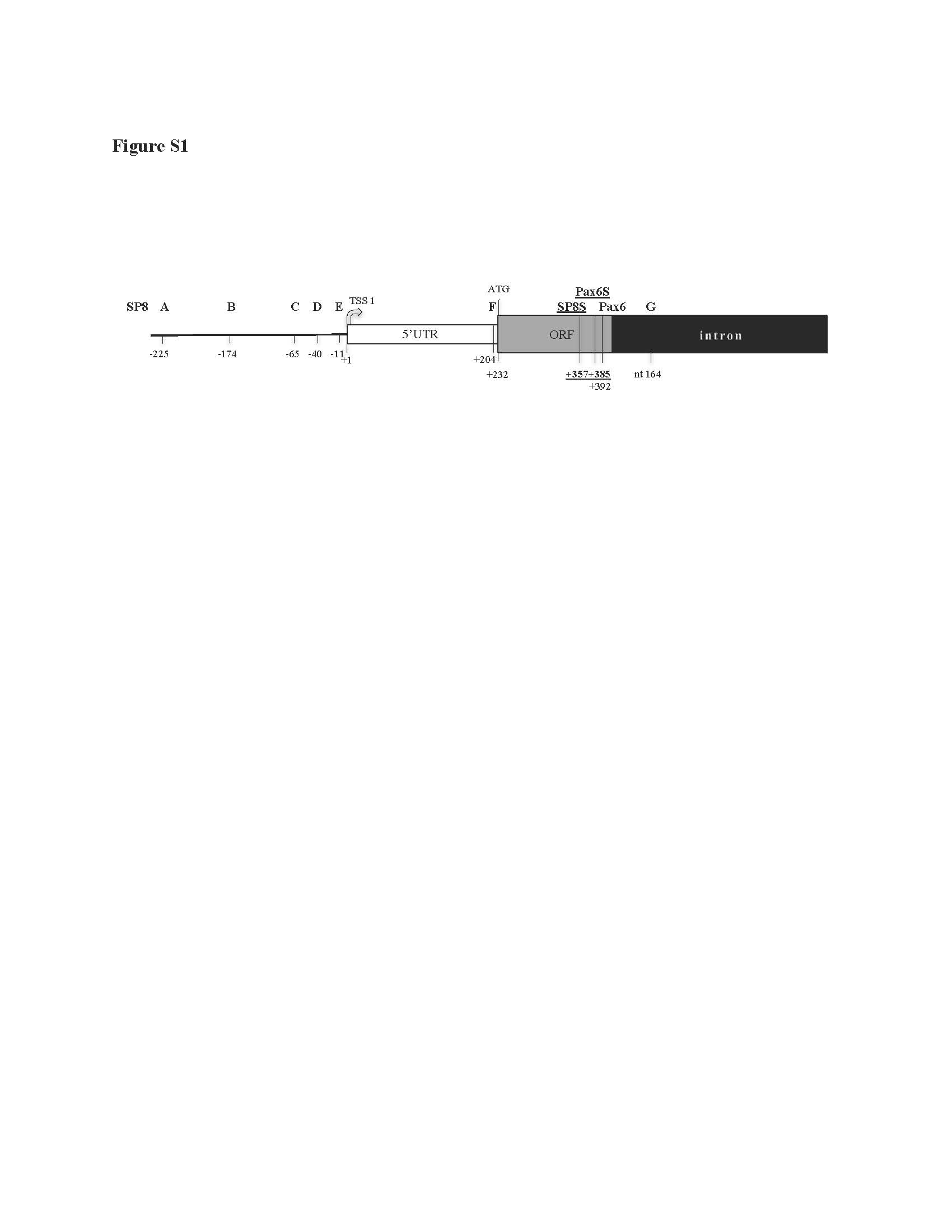

Supplement: Figure S1 — Predicted SP8 binding sites on Ccnd1 Ex1 fragment. The predicted SP8 sites from Table S4 (A–G), the SP8 summit (SP8S), the PAX6 summit (PAX6S), the predicted PAX6 binding site, and the ATG are indicated. The nt positions refer to the Ccnd1 TSS1; SP8 (G) site position is indicated as 164 nt downstream of the exon 1 ORF. The SP8 A-E sites are located in the promoter region; SP8 (F,G) sites are the closest to the SP8 summit. Positions of the SP8S and PAX6S are indicated in bold and underlined. [file Image1.JPEG]

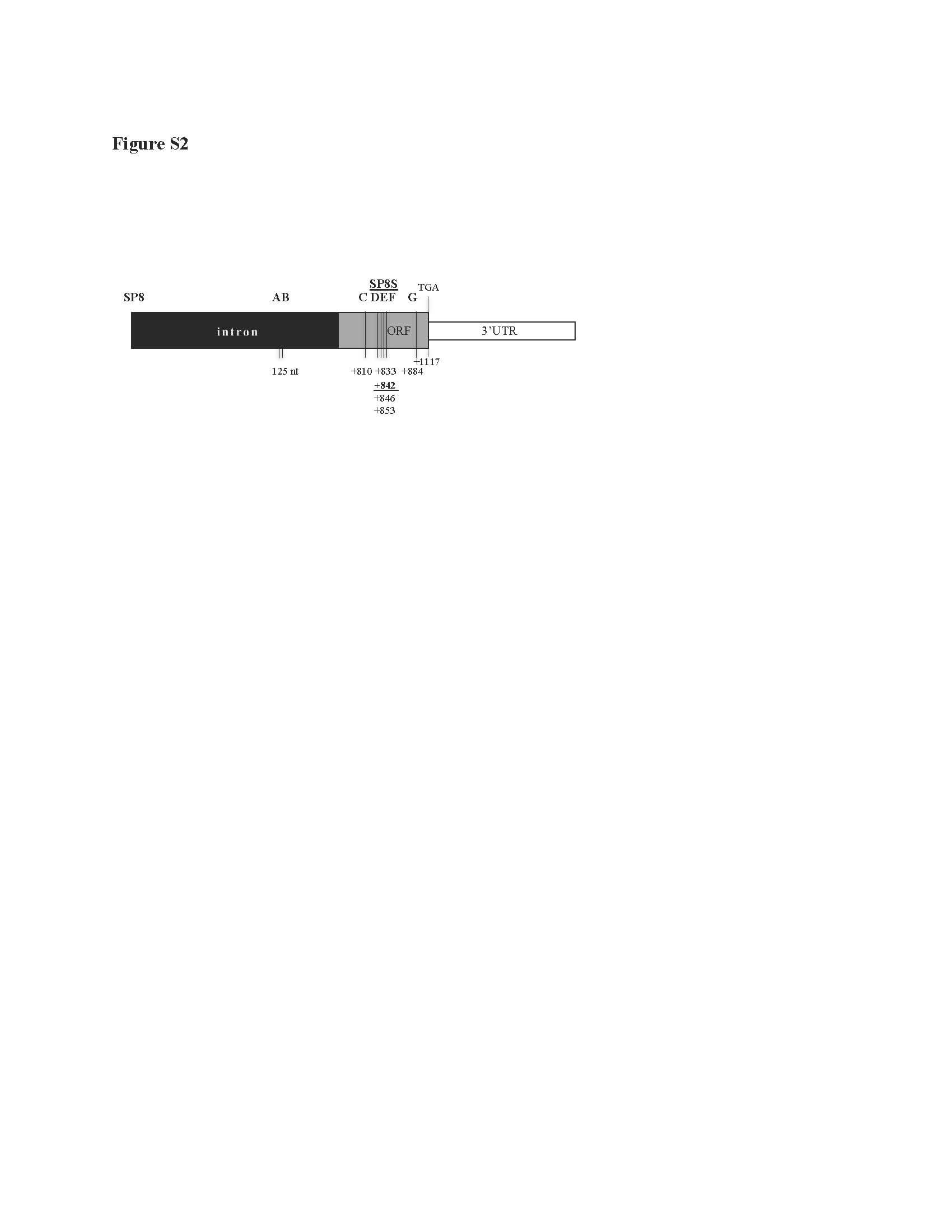

Supplement: Figure S2 — Predicted SP8 binding sites on Ccnd1 Ex5 fragment. The 7 predicted SP8 sites from Table S2 (A–G), the SP8 summit (SP8S), and the stop codon (TGA) are indicated. The nt positions refer to the Ccnd1 TSS1; SP8 (A,B) position is indicated as 125 nt upstream of the exon 5 ORF. Position of the SP8S is indicated in bold and underlined. [file Image2.JPEG]

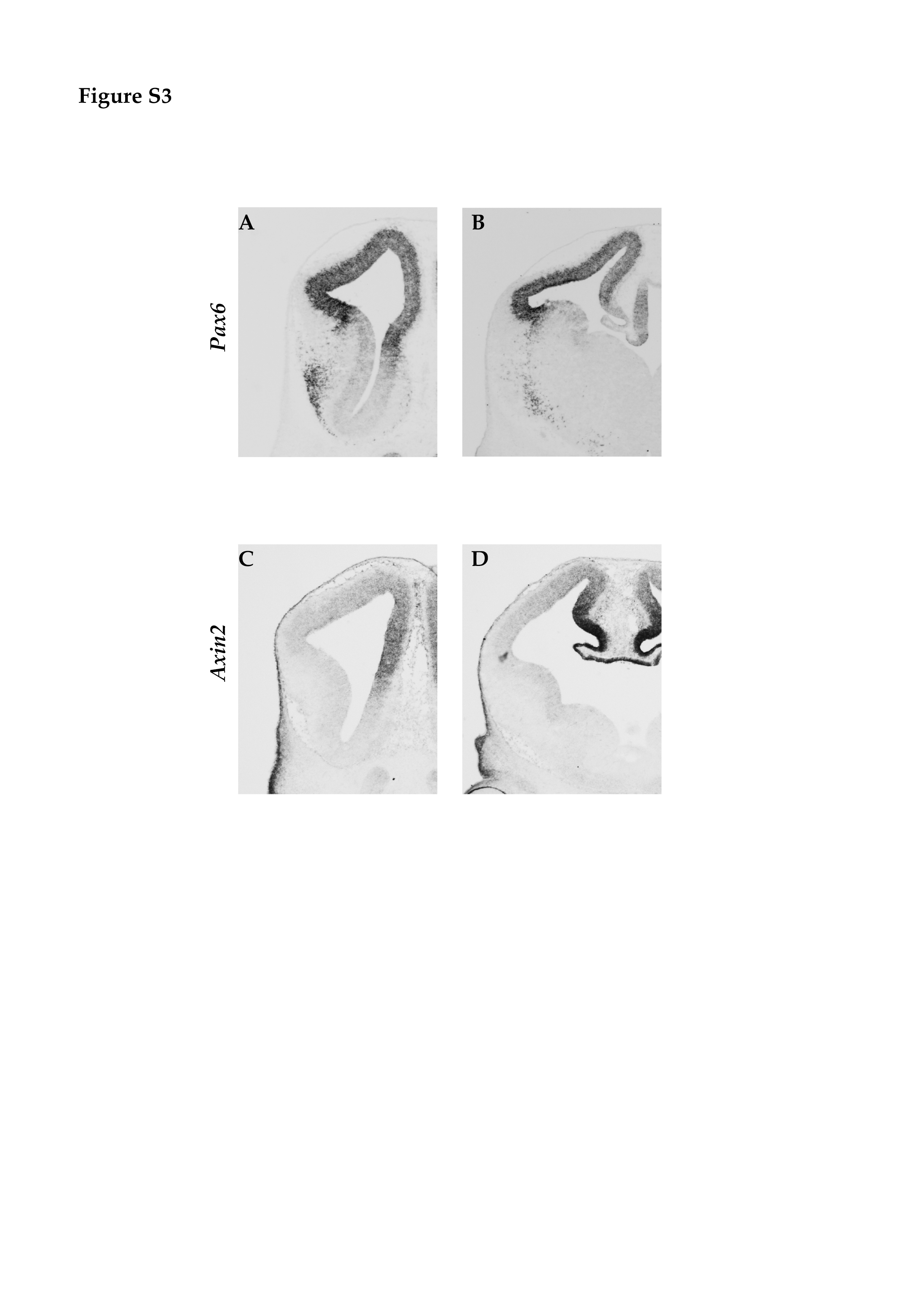

Supplement: Figure S3 — Pax6 and Axin2 expression at E12.5. ISH on E12.5 mouse forebrain coronal sections. Panel (A) shows Pax6 expression in the rostral forebrain and panel (B) shows Pax6 expression in a more caudal section, panel (C) shows Axin2 expression, as proxy of the Wnt pathway activity, in the rostral forebrain and panel (D) shows Axin2 expression in a more caudal section. Bar in (A): 200 μm. [file Image3.JPEG]

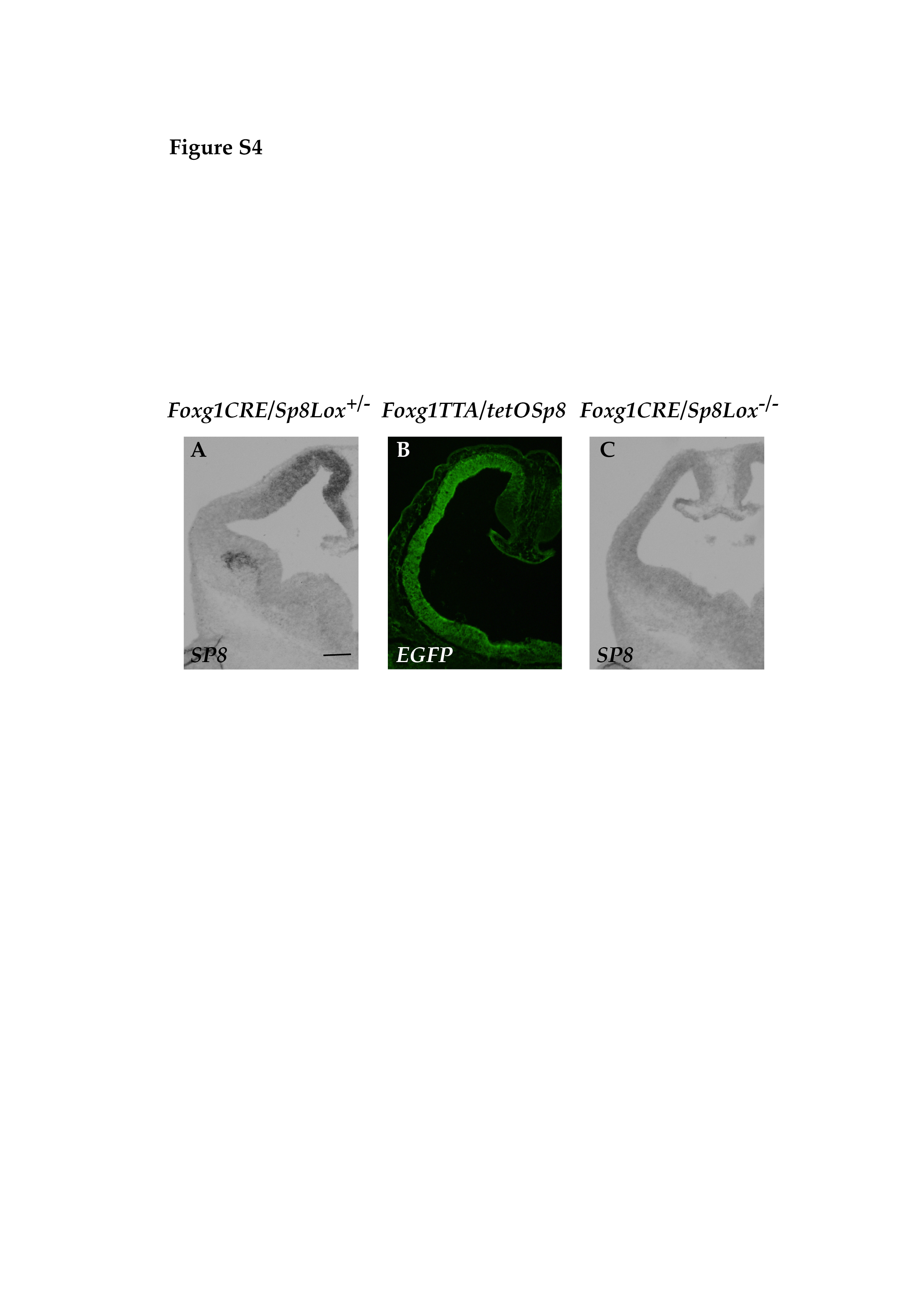

Supplement: Figure S4 — Sp8 expression analysis in the Sp8 LOF and GOF mutants at E12.5. E12.5 mouse forebrain coronal sections. Sp8 mRNA expression levels are shown in the control (A) and Sp8 LOF mutant (C), immunofluorescence of EGFP (B) is shown as a proxy of Sp8 overexpression in the GOF mutant (Borello et al., 2014). Bar in (A): 200 μm. [file Image4.JPEG]

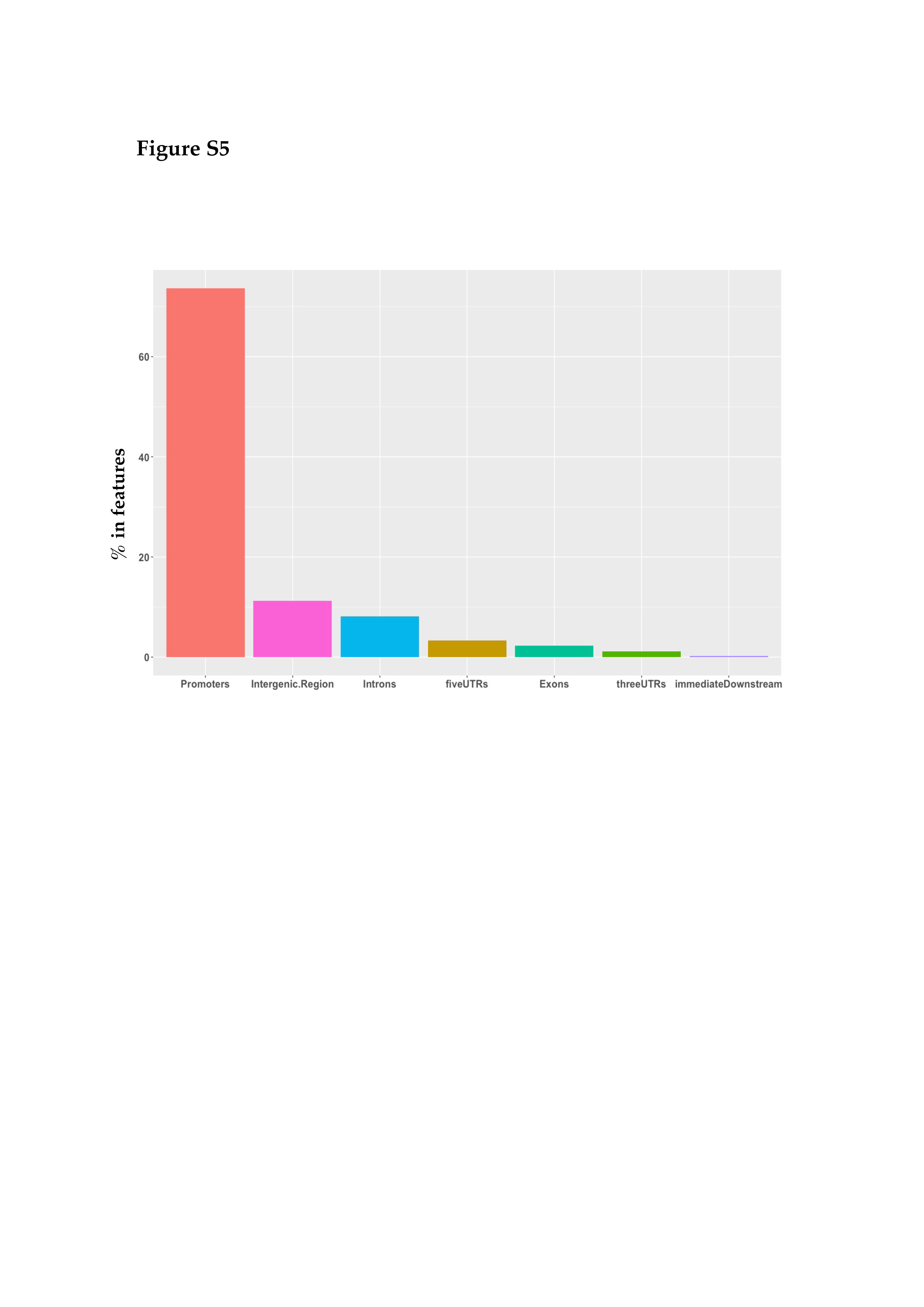

Supplement: Figure S5 — Genome-wide distribution of the SP8 binding sites on gene features. Plot showing the percentage of the SP8 binding sites distributed genome-wide on gene features. [file Image5.JPEG]
