# Supplementary material for: SP8 Transcriptional Regulation of Cyclin D1 During Mouse Early Corticogenesis
Source: Front Neurosci. 2018 Mar 2;12:119. doi: 10.3389/fnins.2018.00119 (PMC5863514; doi:10.3389/fnins.2018.00119)
Supplement: Table S1 — SP8 and H3K27ac ChipSeq fragments identified on the Ccnd1 locus. MACS results of the SP8 and H3K27ac ChipSeq peak calling. Position of the peaks summits is indicated. The column “name” indicates the genomic fragment names used in this study. The fragment named Ex1.2.3 in the H3K27ac ChipSeq dataset contains Ccnd1 promoter, 5′UTR, and exons 1–3; fragment Ex5 contains Ccnd1 exon 5 and 3′U TR. [file Table1.PDF]

Table S1

SP8 ChipSeq with C-18 anti-SP8 antibody

name	chr	start	end	length	summit	tags	-10*LOG10(pvalue)	fold_enrichment	FDR(%)
Ex5	7	152118060	152119107	1048	523	76	405.81	28.43	10.00
Ex4	7	152119606	152120239	634	420	54	327.86	19.48	7.14
Ex2.3	7	152123010	152124315	1306	949	131	850.43	28.95	33.33
Ex1	7	152124905	152126129	1225	569	87	291.02	15.70	5.56

H3K27ac ChipSeq

name	chr	start	end	length	pileup	-LOG10(pvalue)	fold_enrichment	-LOG10(qvalue)
Ex5	7	152118448	152118679	232	13.63	10.20993	2.96774	9.47283
Ex1.2.3	7	152123119	152129310	6192	42.49	41.02037	8.64459	39.44811

SP8 ChipSeq with ab739494 anti-SP8 antibody

name	chr	start	end	length	summit	tags	-10*LOG10(pvalue)	fold_enrichment	FDR(%)
Ex5	7	152118121	152118965	845	414	47	216.91	18.53	9.30
Ex4	7	152119746	152120388	643	225	34	155.20	11.99	9.76
Ex2.3	7	152122724	152124312	1589	1121	57	170.83	11.44	7.46
Ex1	7	152125024	152126130	1107	606	52	129.01	9.51	7.27
