# Supplementary material for: SP8 Transcriptional Regulation of Cyclin D1 During Mouse Early Corticogenesis
Source: Front Neurosci. 2018 Mar 2;12:119. doi: 10.3389/fnins.2018.00119 (PMC5863514; doi:10.3389/fnins.2018.00119)
Supplement: Table S2 — Bioinformatic analysis using the Jaspar software (Mathelier et al., 2016) of the Ccnd1 Ex5 fragment. Position of the predicted SP8 sites refers to the Ex5 fragment full sequence, nt 1-889. [file Table2.DOC]

| 7 putative sites were predicted with these settings (80%) in sequence named **Ccnd1_Ex5 fragment** | | | | | | | |
| --- | --- | --- | --- | --- | --- | --- | --- |
| **Model ID** | **Model name** | **Score** | **Relative score** | **Start** | **End** | **Strand** | **predicted site sequence** |
| MA0747.1 | SP8 A | 7.141 | 0.814772113005646 | 301 | 312 | -1 | gccacccctaaa |
| MA0747.1 | SP8 B | 6.600 | 0.804718013173284 | 313 | 324 | -1 | gaaactccccaa |
| MA0747.1 | SP8 C | 8.422 | 0.838578585991331 | 481 | 492 | 1 | gccaggcccagc |
| MA0747.1 | SP8 D | 7.487 | 0.821202276669227 | 504 | 515 | 1 | cccaaggccact |
| MA0747.1 | SP8 E | 8.354 | 0.837314854404269 | 517 | 528 | -1 | ccccctcctcct |
| MA0747.1 | SP8 F | 6.696 | 0.806502104825607 | 524 | 535 | -1 | tccacttccccc |
| MA0747.1 | SP8 G | 8.807 | 0.845733536888668 | 555 | 566 | 1 | tgcacgcccacc |

**Table S2**
