# Supplementary material for: SP8 Transcriptional Regulation of Cyclin D1 During Mouse Early Corticogenesis
Source: Front Neurosci. 2018 Mar 2;12:119. doi: 10.3389/fnins.2018.00119 (PMC5863514; doi:10.3389/fnins.2018.00119)
Supplement: Table S3 — Ccnd1 expression levels in the Sp8 GOF and LOF mutants. Results of RNASeq analysis on Sp8 mutants obtained with DESeq2 (Love et al., 2014). [file Table3.PDF]

Table S3

***Sp8* GOF**

Ensembl ID	baseMean	log2FoldChange	lfcSE	stat	pvalue	padj	symbol
ENSMUSG00000070348	3587.031184	1.58896041	0.14929722	10.64293369	1.88E-26	1.06E-24	*Ccnd1*

***Sp8* LOF**

Ensembl ID	baseMean	log2FoldChange	lfcSE	stat	pvalue	padj	symbol
ENSMUSG00000070348	2823.489285	-0.267532694	0.091714	-2.91703224	0.003533792	0.09399113	*Ccnd1*
