# Supplementary material for: SP8 Transcriptional Regulation of Cyclin D1 During Mouse Early Corticogenesis
Source: Front Neurosci. 2018 Mar 2;12:119. doi: 10.3389/fnins.2018.00119 (PMC5863514; doi:10.3389/fnins.2018.00119)
Supplement: Table S4 — Bioinformatic analysis of the Ccnd1 Ex1 fragment using the Jaspar software (Mathelier et al., 2016). Position of the predicted SP8 sites refers to the Ex1 fragment full sequence, nt 1-1249. In bold are indicated the SP8 predicted sites in the Ccnd1 promoter (SP8 A–E) showing the highest score values, and the two SP8 sites (F,G) close to the position of the SP8 summit. The position of the predicted PAX6 site is indicated. [file Table4.DOC]

| 29 putative sites were predicted with these settings (80%) in sequence named **Ccnd1_Ex1 fragment** | | | | | | | |
| --- | --- | --- | --- | --- | --- | --- | --- |
| **Model ID** | **Model name** | **Score** | **Relative score** | **Start** | **End** | **Strand** | **predicted site sequence** |
| MA0747.1 | **SP8 A** | **9.241** | **0.853799117900211** | **69** | **80** | **1** | **gccactacccca** |
| MA0747.1 | SP8 | 7.331 | 0.818303127734202 | 75 | 86 | 1 | accccacctcca |
| MA0747.1 | SP8 | 7.334 | 0.818358880598337 | 77 | 88 | 1 | cccacctccaca |
| MA0747.1 | SP8 | 6.660 | 0.805833070455986 | 118 | 129 | 1 | gcccccctcccc |
| MA0747.1 | **SP8 B** | **11.562** | **0.896933250452728** | **120** | **131** | **1** | **ccccctccccct** |
| MA0747.1 | SP8 | 8.092 | 0.83244577093647 | 195 | 206 | -1 | acccttccccct |
| MA0747.1 | SP8 | 8.648 | 0.842778635089508 | 215 | 226 | 1 | gccccccccccg |
| MA0747.1 | SP8 | 8.067 | 0.831981163735345 | 216 | 227 | 1 | ccccccccccgc |
| MA0747.1 | SP8 | 7.778 | 0.826610304490331 | 217 | 228 | 1 | cccccccccgcc |
| MA0747.1 | SP8 | 8.427 | 0.838671507431556 | 221 | 232 | 1 | cccccgccgccc |
| MA0747.1 | SP8 | 7.316 | 0.818024363413526 | 224 | 235 | 1 | ccgccgcccccc |
| MA0747.1 | SP8 | 9.253 | 0.854022129356751 | 226 | 237 | 1 | gccgcccccccc |
| MA0747.1 | **SP8 C** | **12.912** | **0.92202203931352** | **229** | **240** | **1** | **gcccccccccct** |
| MA0747.1 | SP8 | 8.863 | 0.846774257019189 | 230 | 241 | 1 | cccccccccctc |
| MA0747.1 | SP8 | 6.540 | 0.803602955890582 | 231 | 242 | 1 | ccccccccctca |
| MA0747.1 | SP8 | 6.504 | 0.802933921520961 | 248 | 259 | 1 | cccgagccccct |
| MA0747.1 | SP8 | 7.320 | 0.818098700565707 | 253 | 264 | 1 | gccccctccccc |
| MA0747.1 | **SP8 D** | **11.562** | **0.896933250452728** | **254** | **265** | **1** | **ccccctccccct** |
| MA0747.1 | SP8 | 7.903 | 0.82893334049596 | 277 | 288 | 1 | ggccctccccct |
| MA0747.1 | **SP8 E** | **11.562** | **0.896933250452728** | **283** | **294** | **1** | **ccccctccccct** |
| MA0747.1 | **SP8 F** | **6.951** | **0.81124109827709** | **498** | **509** | **1** | **accacagccctc** |
| MA0069.1 | Pax6 | 11.577 | 0.848917153688631 | 686 | 699 | -1 | tttccgcatggatg |
| MA0747.1 | **SP8 G** | **6.416** | **0.801298504172999** | **888** | **899** | **-1** | **ccaactcccaaa** |
| MA0747.1 | SP8 | 8.315 | 0.836590067170512 | 934 | 945 | -1 | acccctccccga |
| MA0747.1 | SP8 | 6.744 | 0.807394150651769 | 1070 | 1081 | -1 | ccctcccccctt |
| MA0747.1 | SP8 | 7.158 | 0.815088045902412 | 1071 | 1082 | -1 | cccctcccccct |
| MA0747.1 | SP8 | 10.938 | 0.885336654712629 | 1072 | 1083 | -1 | ccccctcccccc |
| MA0747.1 | SP8 | 7.320 | 0.818098700565707 | 1073 | 1084 | -1 | gccccctccccc |
| MA0747.1 | SP8 | 9.342 | 0.855676130992759 | 1199 | 1210 | -1 | gccctgccccca |

**Table S4**
